# Synthesis and Investigation of Thermo-Induced Gelation of Partially Cross-Linked Poly-2-isopropyl-2-oxazoline in Aqueous Media

**DOI:** 10.3390/polym12030698

**Published:** 2020-03-21

**Authors:** Alina Amirova, Serafim Rodchenko, Mikhail Kurlykin, Andrey Tenkovtsev, Illia Krasnou, Andres Krumme, Alexander Filippov

**Affiliations:** 1 Institute of Macromolecular Compounds of the Russian Academy of Sciences, Bolshoy pr., 31, Saint Petersburg 199004, Russia; srfm.rodchenko@gmail.com (S.R.); mike_x@mail.ru (M.K.); avt@hq.macro.ru (A.T.); afil@imc.macro.ru (A.F.); 2 Department of Materials and Environmental Technology, Tallinn University of Technology, Ehitajate tee 5, Tallinn 19086, Estonia; illia.krasnou@taltech.ee (I.K.); andres.krumme@taltech.ee (A.K.)

**Keywords:** polyoxazolines, cross-linking polymers, thermo-induced gelation, steady-state flow rheology, turbidimetry

## Abstract

Water-soluble, partially cross-linked poly-2-isopropyl-2-oxazoline combining the properties of chemical and physical gels was synthesized by a two-step procedure. Thermally induced sol-gel transition in its aqueous solution was studied by rheology, light scattering, and turbidimetry. It was demonstrated that the synthesized product is bimodal; it consists of linear and cross-linked components. The cross-linked components are responsible for the gelation, while the linear ones abate the viscosity growth. Heating the solution above the phase transition temperature leads to the self-assembly of the particles into a physical gel. The combination of chemical and physical cross-linking was found to be a prospective route for thermosensitive gel development.

## 1. Introduction

In situ gelling polymer systems, including thermo-gels, find wide application in protein and drug delivery, tissue engineering, cell encapsulation, magnetic resonance imaging contrasting, 3D live cell imaging, etc. [1,2,3,4]. Several factors are considered to initiate the sol-gel transition: the thermally induced chemical cross-linking of macromolecules, physical entanglements between the macromolecular chains, the vitrification of polymers below the glass transition temperature, the phase separation of the solution components, and the crystallization of polymers [5]. Concerning the first two factors, chemical and physical gels are recognized as two peculiar types. Chemical gels are polymers cross-linked by covalent bonds and are able to swell in a large amount of solvent [6,7]. The covalent cross-linking makes the gelation irreversible and the chemical gels elastic. Solvophobic ionic interactions or hydrogen bonding between polymer fragments can cause the formation of physical gels. These interactions are always reversible, therefore physical gels can be easily modified and reshaped. In addition, no cross-linker chemicals are needed to form junctions in physical gels. A combination of physical and chemical cross-linking utilizes the advantages of both polymer gel types and expands their possible application.

Poly(ethylene glycol), Pluronics, poly(*N*-isopropylacrylamide), and chitosan are the most common polymers used for thermo-induced sol-gel transition exploration [8,9,10,11,12,13,14]. Polyoxazoline-based gels have great prospects to be used in biomedical applications due to the nontoxicity and biocompatibility of these polymers [9,15,16]. In general, there are two routes to cross-linked polyoxazoline production, which are the simultaneous copolymerization of monomers with a multifunctional cross-linker [17,18,19,20] and oligo- and polyoxazoline chain cross-linking [21,22,23,24,25,26]. The hydrogels described in literature are characterized by high affinities for water and swelling degrees. At the same time, linear polyoxazolines are known for an ability to form true solutions in water. Thus, there are strong interests in and an insistent need to produce water soluble polyoxazoline with the combination of chemical and physical gel properties, similar to those reported in other types of thermoresponsive polymers [27,28].

A novelty of the approach proposed in this paper is a two-step procedure that involves the synthesis of linear oligomers via the polymerization of 2-isopropyl-2-oxazoline at the first stage and the cross-linking of them with bis-oxazoline at the second one. Three-dimensional objects with the desired properties could be made by the adjustment of the conversion rate and the cross-linker content. The aim of the present work is the synthesis of the cross-linked poly-2-isopropyl-2-oxazoline (CL-PiPrOx) and the study of its gelation in an aqueous solution by means of light scattering and rheology. One of the main tasks is to demonstrate that two-step synthesis could be used to prepare the water-soluble cross-linked poly-2-isopropyl-2-oxazoline, which is capable of thermo-induced gelation in aqueous solutions.

## 2. Materials and Methods

### 2.1. Methods

#### 2.1.1. Synthesis of Cross-Linker

**Figure 1:**
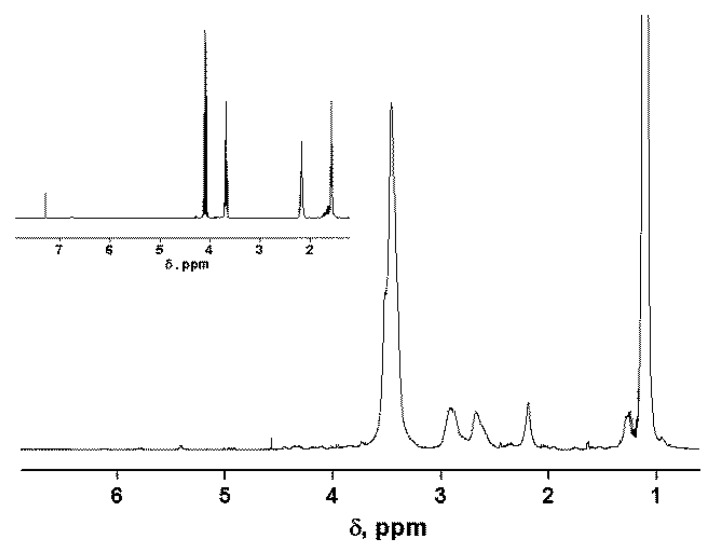
^1^H NMR-spectra of cross-linked PiPrOx. Inset: ^1^H NMR-spectra of bis-oxazoline.

The bis(2-oxazoline) cross-linker, 2,2’-tetramethylen-bis(2-oxazoline), was prepared according to the general procedure described by Witte [29]. Briefly, the mixture of adipodinitrile (11 g, 0.1 mol), 2-aminoethanol (13.5 g, 0.22 mol), and Cd(OCOCH_3_)_2_ (2.3 g, 0.01 mol) was heated at 140 °C for 24 h. The target product was distilled, dried over sodium and redistilled. The yield was 10.5 g (58%), b.p. 123–124 °C (2 mm, Hg), m.p. 44 °C. ^1^H nuclear magnetic resonance (NMR) (δ, ppm, inset in Figure 1): 1.69 (m, 2H) 2.37 (m, 2H) 3.85 (t, 2H) 4.20 (m, 2H). 

#### 2.1.2. Synthesis of Cross-Linked Polyoxazoline

**Scheme 1:**
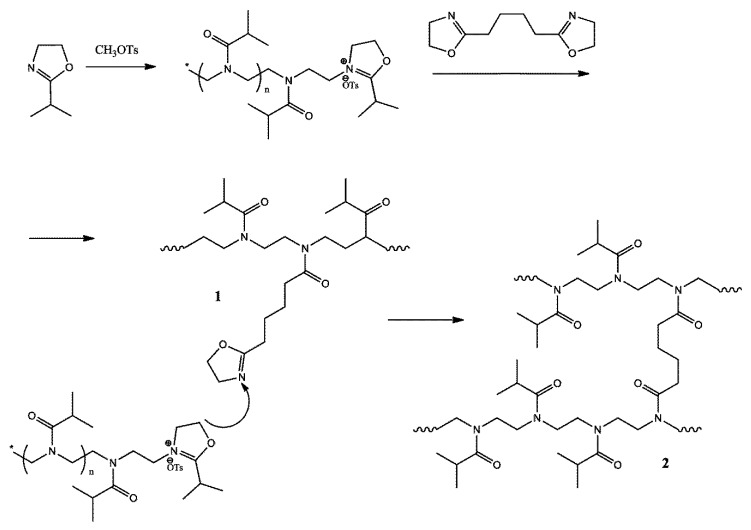
The synthesis of linear (**1**) and cross-linked (**2**) poly-2-isopropyl-2-oxazoline.

The cross-linked polyoxazoline (CL-PiPrOx, Scheme 1) was prepared by the copolymerization of 2-isopropyl-2-oxazoline with 2,2’-tetramethylen-bis(2-oxazoline) in acetonitrile, using methyl 4-toluensulfonate (MeOTs) as an initiator. For the preparation of CL-PiPrOx with 2.5 mol% of cross-linker, the mixture of MeOTs (27.9 mg, 0.15 mmol), isopropyloxazoline (500 mg, 4.42 mmol) and acetonitrile (0.5 ml) was polymerized in a sealed ampoule for 72 h at 70 °C. Then the ampoule was opened and sealed again after the addition of bis(2-oxazoline) (21.6 mg, 0.11 mmol); the polymerization was continued for an additional 24 h at 70 °C. A yellowish product was dialyzed against water and freeze-dried. ^1^H NMR (δ, ppm, Figure 1): 3.5 (m, 4H), 2.6–3.0 (wide d, 1H), 1.1 (wide s, 6H).

#### 2.1.3. Determination of Molecular Weight *R_h-D_* and Hydrodynamic Characteristics

The molecular weight and hydrodynamic characteristics of CL-PiPrOx were determined by static and dynamic light scattering at 21 °C in 2-nitropropane (2-NP), in which no supramolecular structures could be formed. Hydrodynamic radii of scattering objects *R_h-D_*(*c*) at certain concentrations were calculated from values of the diffusion coefficient *D*_0_ using the Stokes–Einstein equation

**Figure 2:**
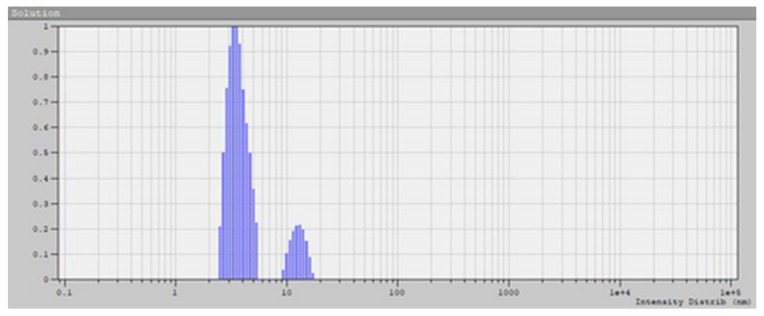
The distribution of light scattering intensity *I* on hydrodynamic radii *R_h-i_* of scattering species of CL-PiPrOx in 2-NP at concentration *c* = 0.0436 g·mL^−3^.

**Figure 3:**
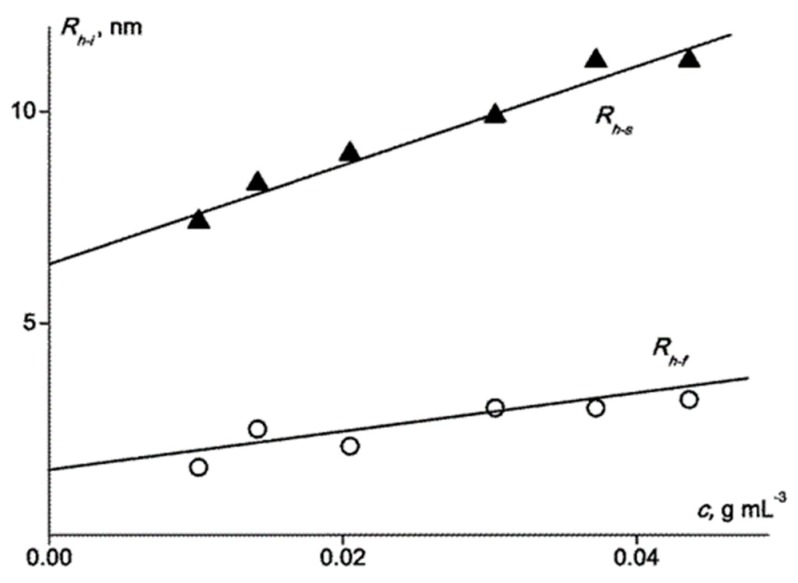
The concentration dependence of hydrodynamic radii of fast *R_f_* and slow *R_s_* modes of CL-PiPrOx in 2-NP.

*R_h-D_* = *k_B_T*/6πη_0_*D*_0_ (1)

where *k_B_* is the Boltzmann’s constant, *T* is the absolute temperature, and η_0_ is the solvent viscosity. The bimodal distribution of light scattering intensity (*I*) on hydrodynamic radii was observed at each concentration (Figure 2). The hydrodynamic dimensions (*R_h-D_*) were determined by the extrapolation of *R_h-D_*(*c*) to *c* = 0 (Figure 3). The weight-average molecular weights (*M_w_*) of scattering objects were obtained by the Debye method, taking into consideration the sample bimodality because of the low asymmetry of light scattering. The contribution *S_i_* of each mode to the summary scattering intensity was estimated from the area under the curve of *I* on the *R_h_* distribution peak (Figure 2). The intensities *I_i_* of light scattered by fast and slow mode particles were calculated from the summary intensity *I* and *S_i_* values as *I_i_* = *I* × *S_i_*. The relative concentrations *c_i_* of fast and slow mode particles, i.e., their ratio, were calculated from the *I_i_* and *R_h-D_* values

*I_i_* ~ *c_i_(R_h__-__D_)_i_^x^* (2)

where the exponent *x* depends primarily on the shape of the scattering particles. The weight concentrations *c_w-i_* = *c × c_i_* of both types of particles were recalculated from the relative concentrations *c_i_* and the polymer weight concentration *c* in the prepared solution. The weight-average molecular weights (*M_w-i_*) of fast and slow mode particles were calculated by the Debye equation

(3) $$\frac{c_{w-i}H}{I_{i}}=\frac{1}{P\left(90^{o}\right)M_{W-i}}+2A_{2}c_{w-i}$$

where *H* is the optical constant

(4) $$H=\frac{{4\pi }^{2}n_{0}^{2}{(dn/dc)}^{2}}{N_{A}\lambda^{4}}$$

where *A*_2_ is the second virial coefficient, *P*(90°) = 1 is the particle scattering factor, *N_A_* is Avogadro’s number, *n*_0_ = 1.3943 mL^3^·g^−1^ is the refractive index of the solvent, λ = 659.1 nm is the incident wavelength, and *dn*/*dc* = 0.128 mL^3^·g^−1^ is the refractive index increment.

#### 2.1.4. Thermoresponsive Behavior Study

CL-PiPrOx was investigated in aqueous solutions; the concentration ranged from 5 to 20 wt. % on heating by light scattering, turbidimetry, and rheology. In light scattering and turbidimetry experiments, the solution temperature (*T*) was changed gradually over the interval from 15 to 47 °C with accuracy ±0.1 °C. Turbidimetry experiments were conducted on solutions equilibrated to the temperature at each step until the transmittance reached the plateau. Details of the experiment are described elsewhere [30]. The measurements of the dynamic regime were carried out during continuous heating at the rates of 0.25, 0.5 and 1 °C per minute. 

Rheological experiments have been done in conditions far away from the glass transition of polymers. The flow behaviour was studied by a shear rate ramp from 0.01 to 100 s^−1^ at temperatures below and above the gel point. The controlled shear rate test was performed within the linear viscosity region at 100 s^−1^ shear rate to study the effect of the temperature on the viscosity of solutions; the heating rate was 0.5 °C min^−1^. The study of gel behavior in a steady-state rotational flow allows the avoidance of complicated elastic effects in non-Newtonian liquids. A sol-gel transition temperature was obtained from oscillation tests by the measurement of storage and loss moduli upon heating from 30 to 50 °C at the heating rate of 0.5 °C·min^-1^. The oscillation frequency was set at a constant of 1 Hz and the strain amplitude at 1%.

### 2.2. Materials and Instruments

^1^H NMR spectra were recorded by an AC400 spectrometer (400 MHz) (Bruker, Billerica, MA, US) for solutions in chloroform. Dialysis was performed using CellaSep (Orange Scientific, Braine-l’Alleud, Belgium) dialysis bags with MWCO3500 D. The chromatographic analysis was carried out by a LC-20AD chromatograph (Shimadzu, Kyoto, Japan) equipped with a TSKgel G5000HHR column 5 μm, 7.8 mm × 300 mm (Tosoh Bioscience, Tokyo, Japan) and refractometric, LS and UV detectors. A LiBr solution (0.1 mol L^−1^) in DMF at 60 °C was used as a mobile phase, which prevents H-bonding in polymer solutions. The calibration was performed relative to poly(ethylene glycol) standards (*M_w_* = 600–40,000). Chemicals and solvents (all Sigma Aldrich, St. Louis, MO, USA) were purified according to usual procedures.

Light scattering and turbidimetry experiments were carried out by a Photocor Complex instrument (Photocor Instruments Inc., Moscow, Russia) equipped with a Spectra-Physics He-Ne laser (wavelength λ = 659.1 nm), a Photocor-FC2 correlator with 288 channels and a Photocor-PD detection device for measuring the transmitted light intensity. The autocorrelation function was processed with a DynaLS soft (ver. 8.2.3, SoftScientific, Tirat Carmel, Israel). Toluene was used as a calibration liquid with an absolute scattering intensity of 8.6 × 10^−6^ mL^−1^. The solutions of polymers in 2-NP were filtered into dust-free vials using Millipore filters with hydrophobic PTFE membranes and pore sizes of 0.45 μm (Merck, Darmstadt, Germany). The refractive index increment was determined by means of an RA-620 refractometer (KEM, Tokyo, Japan).

The flow behavior of the CL-PiPrOx solution in water was studied by an Physica MCR501 rheometer (Anton Paar, Graz, Austria) with plate-plate measuring geometry (43 mm diameter, gap 1 mm). All measurements were carried out under the cover to prevent water evaporation from the solution.

## 3. Results

### 3.1. Synthesis and Characterization

The synthesis of the cross-linked polymers was carried out in two stages. In the first stage, 2-isopropyl-2-oxazoline was polymerized in an acetonitrile solution using methyl toluenesulfonate as an initiator at a 30:1 feed ratio with an up to 75%–80% conversion. The conversion was controlled by NMR using the intensity of isopropyloxazoline signals at 4.01 ppm (oxazoline cycle proton signal at position 5, Figure 1). In the second stage, a certain amount of bis-oxazoline was added to the reaction mixture containing living polyoxazoline chains, after which the polymerization was continued until the monomers were almost completely exhausted.

**Figure 4:**
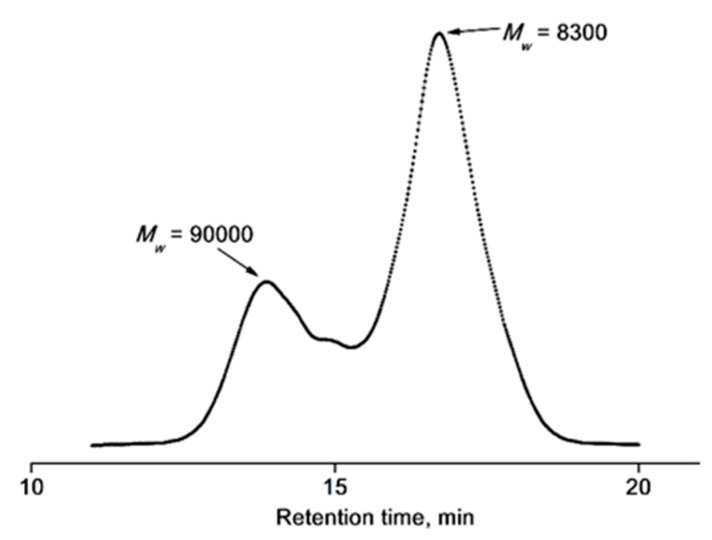
GPC trace for CL-PiPrOx in the DMF and LiBr system.

The gel permeation chromatography (GPC) of CL-PiPrOx shows that the obtained polymer has a bimodal distribution, with the amount of low molecular component **1** at about 70% (Figure 4). As far as GPC is performed in DMF+LiBr solutions, which prevent intermolecular interactions, the bimodality is attributed to two different types of macromolecules synthesized, but not to aggregates of any kind.

The same distribution was detected by the dynamic light scattering (DLS) within the investigated concentration range (Figure 2 and Figure 3). This bimodality of the polymer under question probably reflects the two components’ presence in the synthesized product. The narrow molecular-weight distribution is typical for the cationic polymerization of linear oxazolines in the living chain mode. It is possible to assume that, after the cross-linker addition, one can observe mainly an elongation of the polyoxazoline chains **1** (Scheme 1). There is no cross-linking due to the steric hindrances in the chain, which limit the living terminal group access to another macromolecular coil. As a result, structure **1** appears as a linear chain with the oxazoline moiety of bis-oxazoline cross-linker in the side fragment that was reported by Saegusa for bis-oxazoline to initiator ratio equal to 2.5 [31]. Then, the interaction of this side oxazoline moiety with the living terminal group of another linear macromolecule leads to the formation of object **2**. Taking into account that (i) the product is well-soluble and (ii) cross-linking by bis-oxazoline cannot be ignored, the formation of a partially cross-linked system can be supposed.

Assuming that the linear chains, observed by DLS and termed as the fast mode, are random coils, the second power in Equation (2) is valid [32]. A similar formula is true of cross-linked particles considered as the slow mode with *R_s_* dimensions due to the fractal dimensions of swollen gels [33]. According to these equations, the relative weight concentration of linear component **1** was estimated as 0.73, which is in agreement with the GPC data. The weight-average molecular weights *M_w_* were estimated as 5100 and 116,000 for **1** and **2**, respectively. One can note the divergence between the DLS and GPC data—5100 vs. 8300 (component **1**) and 116,000 vs. 90,000 (component **2**). We presume that GPC, calibrated with poly(ethylene glycol) standards, overestimates the *M_w_* values for our product. In spite of this fact, the *M_w_* of the cross-linked component estimated by GPC is lower than that estimated by light scattering, which is explained by the higher intramolecular density and the lower hydrodynamic volume. In both cases, the *M_w_* value of component **2** exceeds one for a hypothetical star, with a bis-oxazoline core and four arms of component **1**. On the other hand, it seems incorrect to compare component **2** to hypebranched polymers, because the latter demands using AB_n_-type monomers and the polymerization process according to Flory [34]. Thus, we suppose that component **2** is a soluble and partially cross-linked poly-2-isopropyl-2-oxazoline in which the number of cross-linking points is not enough to achieve the hydrogel form. The designed length between the knots in the target network was estimated at 24–26 monomer units, based on the monomer-to-initiator feed ratio of 30:1 up to the conversion of 80% and the following cross-linking of the oligomers by bis-oxazoline. Exactly this sparse network provides solubility to the synthesized product. Taking into account the mentioned length of oligomers and the *M_w_* of 5100, it can be concluded that component **1** particles contain on average 1.8 bis-oxazoline molecules and a number of cross-linking points.

### 3.2. Thermoresponsive Behavior of CR-PiPrOx

Special approaches are required to study the flow behavior of gelling systems based on linear and branched polymers [35,36] and, in particular, in the vicinity of gel formation [37,38]. Most commonly, a solution of the gelling polymer goes over into an assembly of infinite molecular weight cross-linked particles with significant elasticity [39]. In such systems, at least one of the cross-linked polymer particles has grown to a microscopic size [38]. This growth can be detected reliably by the DLS method.

**Figure 5:**
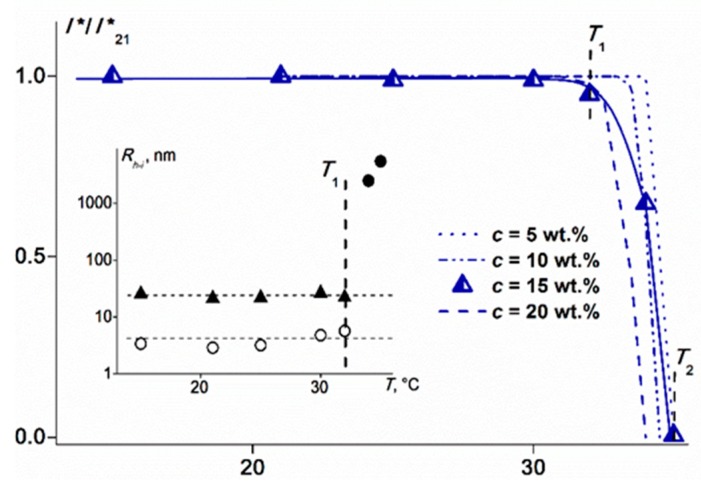
The temperature dependence of the optical transmittance I*, related to its value I*_21_ at *T* = 21 °C, of CL-PiPrOx aqueous solutions. *Inset:* The temperature dependences of hydrodynamic radii of the fast (*R_f_*, open circles) and the slow (*R_s_*, closed triangles) mode particles and aggregates (closed circles) for 15 wt.% CL-PiPrOx in water.

The hydrodynamic dimensions *R_h_* of both modes of CL-PiPrOx in water at *c* = 15 wt. %, observed by DLS at low temperatures, exceeded the corresponding values in 2-nitropropane—namely, *R_f_* = 4.1 nm (in water) and 1.5 nm (in 2-NP), and *R_s_* = 23 nm (in water) and 6.5 nm (in 2-NP) (Figure 3 and Figure 5). This difference can be explained by the fact that: (i) the concentration dependence of the diffusion coefficient as far as the hydrodynamic radii in water were obtained at *c* = 15 wt. %, while the *R_h_* of single macromolecules was extrapolated to zero concentration (Figure 3); (ii) the aggregation could have occurred due to hydrophobic interactions between butylene fragments.

As can be seen from the inset in Figure 5, the radii of the fast and the slow modes in the 15 wt.% solution, in spite of the data spread, were nearly constant up to *T*_1_ = 32 °C, above which particles larger by an order of magnitude appeared. A drastic aggregation of particles began upon the isopropyl-2-oxazoline unit dehydration. The appearance of large objects led to the optical transmittance I* dropping to zero at *T*_2_ = 36 °C (Figure 5). Thus, *T*_1_ and *T*_2_ could be referred to as the onset and the end temperatures of the phase separation process.

**Figure 6:**
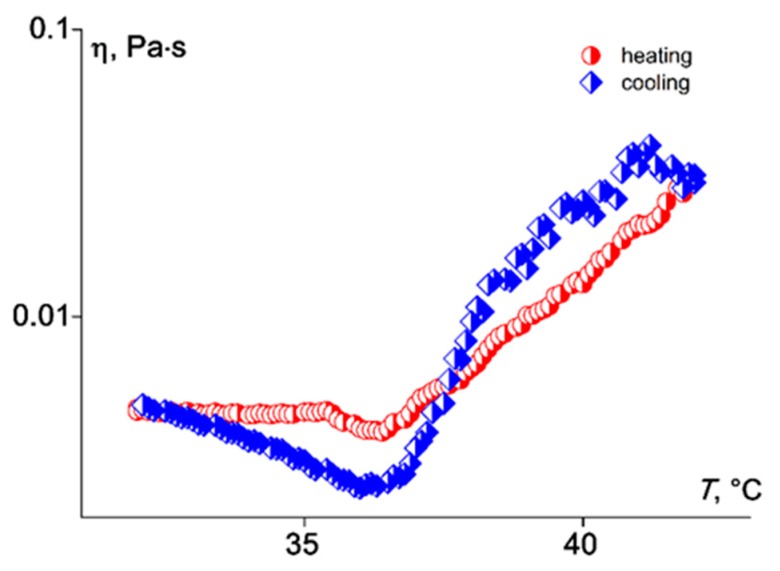
The temperature dependence of the dynamic viscosity η during the temperature sweep experiments in the heating-cooling cycle. The concentration is 15 wt.%; the shear rate γ = 100 s^−1^.

Water molecules formed a hydration layer via hydrogen bonding with the amide and carbonyl groups of each monomer unit. As the solution temperature increased, dehydration of the amide groups started and, at a certain temperature, the chain-to-chain interaction prevailed over the polymer-to-water. This phase separation was found to be reversible [40,41]. However, the long-term exposure above the cloud point (2 hours and more at 65–70 °C) led to an irreversible phase separation due to the isothermal crystallization of the polymer chains [41,42,43]. Figure 6 shows the temperature dependence of the dynamic viscosity of the 15 wt.% solution in a heating-cooling cycle. It shows that the gelation process of this gel is essentially reversible. As the heating rate was 0.5 °C·min^−1^, the experimental processes discussed here took less than 30 minutes within the narrow temperature range near the *T*_1_ and the gelation held off the irreversible crystallization. Moreover, the gel’s stability was checked by a viscosity measurement; at 44 °C, it was constant over one hour.

**Figure 7:**
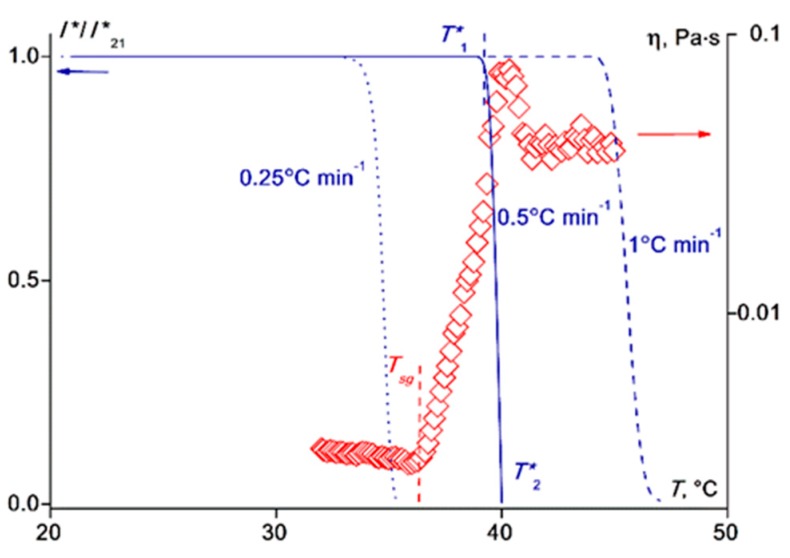
The temperature dependences of the relative optical transmittance (triangles) and the viscosity (lines) of the 15 wt.% CL-PiPrOx aqueous solution obtained at the heating rates 0.5 °C·min^−1^ (triangles and solid line), 0.25 °C·min^−1^ (dot line), and 1.0 °C·min^−1^ (dash line).

The change in the particle sizes affected the solution viscosity. The measurement of the dynamic viscosity in the steady state regime allowed the leveling of the effect of individual cross-linked particles on the gel flow that could interfere with the gelation process examination. The CL-PiPrOx exhibited a temperature-induced sol-gel transition in water; the viscosity η value rose approximately 25 times upon heating (Figure 7).

**Figure 8:**
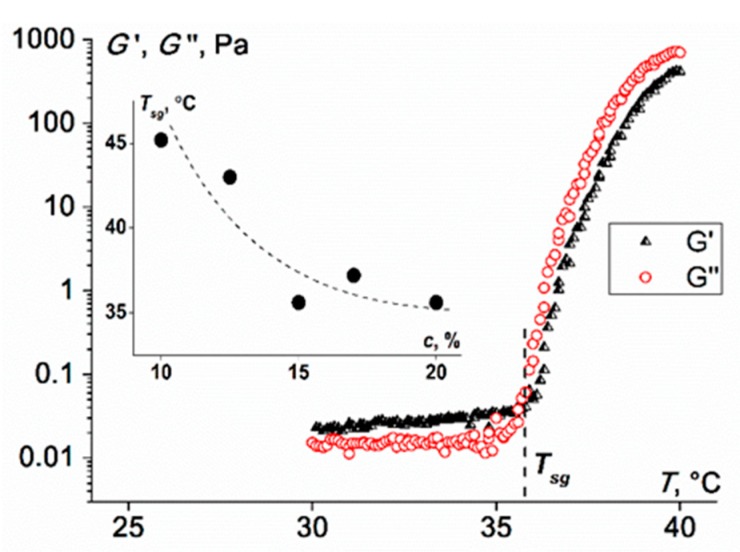
The dependence of storage *G*′ and loss *G*″ moduli on the temperature at the concentration 15 wt.%. *Inset*: the dependence of the sol-gel temperature on the solution concentration.

The sol-gel transition temperature (*T_sg_*) was determined as 36 °C in the oscillation regime at the point where the storage modulus (*G*′) became equal to the loss modulus (*G*″). The measurement of moduli dependence on temperature was performed in the range of 30 to 50 °C at the heating rate of 0.5 °C·min^−1^ (Figure 8). In the temperature region below 36 °C, *G*′ was lower than *G*″, and the storage modulus decreased upon the temperature growth, which is common in liquids. In the region above 36 °C, *G*′ and *G*″ increased dramatically upon heating and *G*′ overcame *G*″, which evidenced the formation of an elastic gel network.

The *T_sg_* versus the solution concentration is depicted in the inset in Figure 8. The *T_sg_* value increases with the solution dilution. A similar tendency was observed in transmittance experiments (Figure 5), however, within a narrower temperature range. Such concentration dependences of the phase transition temperature are typical for thermoresponsive polymers and, in particular, poly-2-alkyl-2-oxazolines [44,45,46,47].

The transition temperatures *T*_sg_ found by the rheometry and the *T*_1_ determined by the turbidimetry for 15 wt.% solutions differ by 4 °C. This could be explained by the variety in experiment conditions: the rheometry was carried out at the heating rate of 0.5 °C per minute, while the temperature in the turbidimetry was changed stepwise with equilibration at each step. To clarify this phenomenon, additional measurements of the optical transmittance at the heating rates of 0.25, 0.5 and 1.0 °C·min^−1^ were carried out (Figure 7). One can see that increasing the heating rate resulted in the over evaluated temperature of the phase transition and a sharper drop in the solution transparency. This confirms the significance of the heating rate in the determination of phase transition temperature. However, the similar heating rate of 0.5 °C·min^−1^ in the rheology and the turbidimetry experiments resulted in a transparency drop at the temperature *T*_1_*, which is close to but higher than the sol-gel point (Figure 7). The visco-elastic behavior is probably more sensitive to the nano-sized aggregate formation [48] than the optical transmittance, which is insensitive to objects with dimensions below the diffraction limit [49].

The viscosity vs. temperature curve has a slight negative slope below the *T_sg_* (Figure 7), which is typical for solutions of linear polymers [50,51]. Above the *T_sg_*, the viscosity curve takes a sharp positive slope. The solution viscosity rise was caused by the self-assembly of polymer particles into aggregates (see the inset in Figure 5) due to the thermo-induced hydrogen and hydrophobic interactions between particles. At the affinity of 40 °C, the curve passes the maximum and levels off. The viscosity drop is observed near 41 °C, but the nature of this phenomenon is not clear. The following plateau in the viscosity curve probably reflects approaching the equilibrium between the solvent viscosity decrease and the aggregate growth upon heating. Similar phenomena were observed for triblock copolymers based on poly(d,l-lactic acid-co-glycolic acid) and poly(ethylene glycol) [52].

**Figure 9:**
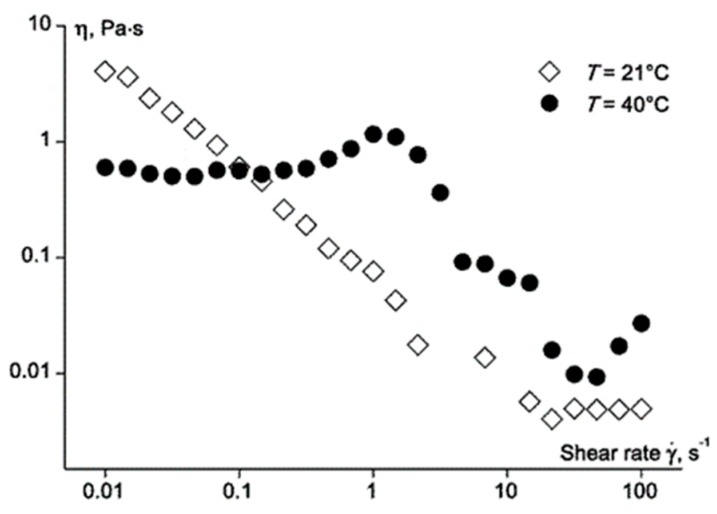
The dependence of the viscosity on the shear rate for 15 wt.% CL-PiPrOx in water at different temperatures.

**Figure 10:**
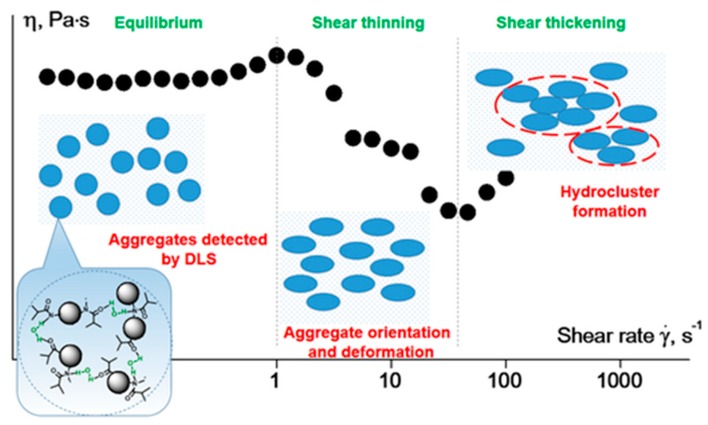
The jamming of hydroclusters in a flow.

The flow behavior of the CL-PiPrOx solutions in water varied at different temperatures (Figure 9). At 21 °C, the solution flowed as a typical viscoelastic fluid displaying the shear thinning effect. Above the *T_sg_*, the solution showed Newtonian flow within the shear rate ranging from 0.01 to 1 s^−1^, and the shear thinning within a 1–100 s^−1^ interval. At lower shear rates, the thermodynamic motion of the gel network, together with the solvation by water, held the system in a dynamic equilibrium with the flow shear. Near $\dot{\gamma }=1$ s^−1^, the viscosity grew and passed over a maximum that corresponded to the greatest strength of the gel. The flow with a shear rate above 50 s^−1^ deformed and oriented aggregates, which caused the viscosity decrease (the shear thinning). At yet higher shear rates, the hydrodynamic interaction—i.e., the more frequent particle collision—in the flow caused the aggregates’ density fluctuation which resulted in hydroclusters’ formation. This retarded the motion of the fluid and led to an abrupt increase in the viscosity (the shear thickening). There are two fundamentally different mechanisms of the hydroclusters’ formation proposed for colloidal systems: jamming when the transient physical network appears between particles and cross-linking when the network junction points’ lifetime is long—for example, the hydrogen bonding between particles [42,53]. The hydrophobic interaction could also cause the supramolecular structure formation, but much weaker [54]. The shear thickening observed at high shear rates resulted from the jamming mechanism, i.e., the orientation and the deformation of aggregates of particles and consequent formation of transient hydroclusters (Figure 10). Gelling via the jamming mechanism was reported for a variety of associative polymers and described in a comprehensive review by Chassenieux and co-workers [55].

## 4. Conclusions

The cross-linked poly-2-isopropyl-2-oxazoline was synthesized by a two-step procedure. First, the synthesis of oligo-2-isopropyl-2-oxazoline chains by cationic ring opening polymerization, and then cross-linking by bis(2-oxazoline).

The bimodality of the sample was detected by GPC and DLS methods and explained by the presence of linear and cross-linked components. They both play significant roles in the thermo-induced gelation upon heating. The cross-linked components, despite being the minority, particularly dominate the thermo-rheological behavior. At the same time, the linear components, which are the majority, moderate the viscosity growth. Therefore, the polymer under investigation can be considered a weak gel with a low amount of junction points.

The reported approach to the synthesis of cross-linked polyoxazolines potentially allows us to vary the average distance between the knots and to obtain more or less “soft” polymer networks with tunable thermo-induced gelation. The influence of the cross-linker content on the polyoxazoline thermo-induced gelation is under investigation and will be reported later.
